# Exploring long COVID condition in Latin America: Its impact on patients’ activities and associated healthcare use

**DOI:** 10.3389/fmed.2023.1168628

**Published:** 2023-04-20

**Authors:** Adriana Angarita-Fonseca, Rodrigo Torres-Castro, Vicente Benavides-Cordoba, Santos Chero, Mauricio Morales-Satán, Bricia Hernández-López, Rafael Salazar-Pérez, Santiago Larrateguy, Diana C. Sanchez-Ramirez

**Affiliations:** ^1^Université du Québec en Abitibi Témiscamingue, Rouyn-Noranda, QC, Canada; ^2^Centre de Recherche du CHU de Québec, Québec City, QC, Canada; ^3^Universidad de Santander, Bucaramanga, Colombia; ^4^Department of Physical Therapy, University of Chile, Santiago, Chile; ^5^Facultad de Ciencias de la Salud, Pontificia Universidad Javeriana Cali, Cali, Colombia; ^6^Universidad Privada Norbert Wiener, Lima, Peru; ^7^Hospital General Docente de Calderón, Quito, Ecuador; ^8^Centro de Enfermedades Respiratorias y Rehabilitación Pulmonar (CERRP), Pachuca, Mexico; ^9^Instituto Profesional en Terapias y Humanidades, Puebla, Mexico; ^10^Universidad Adventista del Plata, Libertador San Martín, Argentina; ^11^Centro Privado de Medicina Respiratoria, Paraná, Argentina; ^12^University of Manitoba, Winnipeg, MB, Canada

**Keywords:** activity limitations, long COVID, healthcare use, long COVID disability, Latin America

## Abstract

**Background:**

Studies exploring long COVID condition (LCC) in low- and middle-income countries are scarce. Further characterization of LCC patients experiencing activity limitations and their associated healthcare use is needed. This study aimed to describe LCC patients’ characteristics, its impact on activities, and associated healthcare use in Latin America (LATAM).

**Participants:**

Individuals who (cared for someone or) had COVID-19 and could read, write, and comprehend Spanish and lived in a LATAM country were invited to complete a virtual survey. Sociodemographic characteristics, COVID-19 and LCC symptoms, activity limitations, and healthcare use.

**Results:**

Data from 2,466 people from 16 countries in LATAM were analyzed (females = 65.9%; mean age of 39.5 ± 53.3 years). 1,178 (48%) of the respondents had LCC symptoms (≥3 months). These were more likely to have COVID-19 earlier in the pandemic, were older, had no COVID vaccines, had more comorbidities, needed supplementary oxygen, and reported significantly more COVID-19 symptoms during the infectious period. 33% of the respondents visited a primary care provider, 13% went to the emergency department, 5% were hospitalized, 21% visited a specialist, and 32% consulted ≥1 therapist for LCC symptoms mainly extreme fatigue, sleep difficulties, headaches, muscle or joint pain, and shortness of breath with activity. The most consulted therapists were respiratory therapists (15%) and psychologists (14%), followed by physical therapists (13%), occupational therapists (3%), and speech pathologists (1%). One-third of LCC respondents decreased their regular activities (e.g., work, school) and 8% needed help with activities of daily living (ADLs). LCC respondents who reduced their activities reported more difficulty sleeping, chest pain with activity, depression, and problems with concentration, thinking, and memory, while those who needed help with ADLs were more likely to have difficulty walking, and shortness of breath at rest. Approximately 60% of respondents who experienced activity limitations sought a specialist and 50% consulted therapists.

**Conclusions and relevance:**

Results supported previous findings in terms of the LCC demographics, and provided insight into LCC impact on patients’ activities and healthcare services used in LATAM. This information is valuable to inform service planning and resource allocation in alignment with the needs of this population.

## Background

The Coronavirus Disease-2019 (COVID-19) continues its spread worldwide, reaching over 754 million confirmed cases, including 6.8 million deaths as of February 7, 2023 (1). As the pandemic progresses, a large subgroup of people with COVID-19 disease reported a wide range of new, recurring, or ongoing health problems or symptoms months after infection, known as long COVID (2). According to the World Health Organization (WHO), long COVID condition (LCC) “occurs in individuals with a history of probable or confirmed SARS-CoV-2 infection, usually 3 months from the onset of COVID-19 with symptoms that last for at least 2 months and cannot be explained by an alternative diagnosis” (3, 4). In early reports, the WHO estimated that at least 10–20% of people had one or more symptoms 12 weeks or more after their initial diagnosis (5). A higher prevalence of LCC has recently been described at 90 and 120 days after infection (32 and 49%, respectively) (6). Overall, the prevalence of LCC has not been determined; wide variations have been reported due to differences in follow-up time, the characteristics of the population studied, and the type of data used (7). Despite the global impact of COVID-19, studies reporting LCC have been conducted primarily in high-income countries, and its effect in low- and middle-income regions has not been widely explored (6–8).

COVID-19 is primarily a respiratory disease, but it can also affect other systems, such as musculoskeletal, cardiovascular, or neurological systems, leading to a wide range of symptoms in the short, medium, and long term in COVID-19 survivors (8–10). The most commonly reported LCC symptoms are chronic fatigue, dyspnea, and brain fog, which in many cases affect the patient’s activities of daily living (ADL) (11, 12), quality of life and the ability to return to work in the active population (8). Although the impact of LCC on patients’ regular activities has been reported, the characteristics of the population more likely to be affected and their healthcare use have not been described. Due to the significant impact of LCC on patients’ lives, the demand for care is expected to increase (13, 14). Therefore, healthcare systems and healthcare providers should aim to improve the diagnosis and management of patients with this condition. Further characterization of the patients experiencing difficulties returning to their regular activities will support this objective.

In the Latin American (LATAM) region, failures of health systems to adequately prevent and control chronic diseases are likely to result in a higher proportion of the population at risk of developing complications related to COVID-19 and LCC (15, 16). Additionally, public health emergencies challenge the ability of the healthcare systems to meet the essential needs of the population, increasing gaps in access and quality of care (16). To the best of our knowledge, there are no studies exploring healthcare use among LCC patients in the LATAM region. Therefore, this study aimed to describe LCC patients’ characteristics, LCC impact on patients’ activities, and associated healthcare use in LATAM.

### Methodology

#### Study design

This study used data collected between 1 November and 1 December 2022, through an online open survey completed by a convenience sample from countries of LATAM. We included individuals residing in any country in LATAM, who reported having COVID-19 and/or cared for someone (e.g., child, parent, etc.) who had COVID-19, and could read, write, and comprehend Spanish. This study was approved by the University of Manitoba ethics committee (HS25587/H2022:230). It followed the Checklist for Reporting Results of Internet E-Surveys (CHERRIES) (17).

#### Recruitment

An electronic questionnaire was widely distributed on multiple sites accessed by a heterogeneous population on social networks (Facebook, LinkedIn, Twitter, WhatsApp, and Instagram) and was sent directly by email to other healthcare providers who were also asked to share it (snowball effect) within their personal and professional networks. The questionnaire was completely anonymous, and no personal identifiers such as names or emails, or IP addresses were collected. Participation was voluntary and non-monetary compensation was offered. Before completing the survey, the participants were informed about the aim of the study, the length of time of the survey, and the voluntary nature of their participation. It was also mentioned that by clicking next and advancing to the next page, they gave their consent to participate in the study.

#### Questionnaire and variables

A survey (25 questions) was developed by the research group based on the lists of main LCC symptoms assessed in the C19-YRS screening tool (18). The usability and technical functionality of the Google forms® (Online survey services) questionnaire were tested by all the co-authors before beginning public distribution of the survey. The online form had 10 pages, and all items on the current page had to be filled to move to the next page. However, “prefer not to answer” was one of the options. Unfortunately, Google Forms does not have a feature to track incomplete surveys. The items were not randomized as the survey had a logical order.

Respondents were able to review and change their answers using the back and forward buttons before submitting the final responses. After the form was submitted, the participant could not make any changes to their answers. However, the survey was immediately available again so that the respondent could fill it out for another family member.

Sociodemographic variables, such as country of residency, age, sex, income, and education, were collected. Questions related to the episode of COVID-19 infection included: date of diagnosis (first episode), COVID-19 confirmed with a test, number of COVID-19 vaccines received, smoking status, comorbidities diagnosed at that time of having COVID-19 (high blood pressure or other circulatory problems, breathing problems, heart problems, diabetes, obesity, kidney problems, others), presence and severity of 18 symptoms, and an open-ended question for other symptoms. Participants were also asked if they or the person they care for had COVID symptoms for ≥3 months after infection (LCC), and further items related to LCC were only displayed based on the affirmative response. People with LCC were asked about the presence and severity of the 18 symptoms and an open-ended question for other symptoms, whether LCC symptoms made them reduce the time spent on their usual activities (e.g., work, study, etc.) or have limited their ADLs (e.g., walking, bathing, showering, dressing, etc.). Participants with self-reported LCC were asked how poor or good they rated their health on that day (0 worst to 10 best possible health). Information about the use of health services due to COVID-19 or LCC was also collected, including visits to a primary care provider (PCP), emergency department (ED), and/or hospital, need for oxygen or other respiratory support, and visits to specialists or therapists.

### Statistical analysis

Participants’ characteristics, symptoms, and healthcare use were presented using mean (standard deviations) and percentages. Comparisons between groups were completed with Chi-Squared or independent t-tests as appropriate. Crude and adjusted logistic regression models were used to explore the characteristics of the participants in relation to reporting LCC, and limitations in their regular activities or ADLs. Statistical significance was accepted at *p* values below 0.05. All analyses were performed using SPSS software, version 28 (IBM Corp., Armonk, NY, USA).

## Results

### Responders of the survey

**Figure 1:**
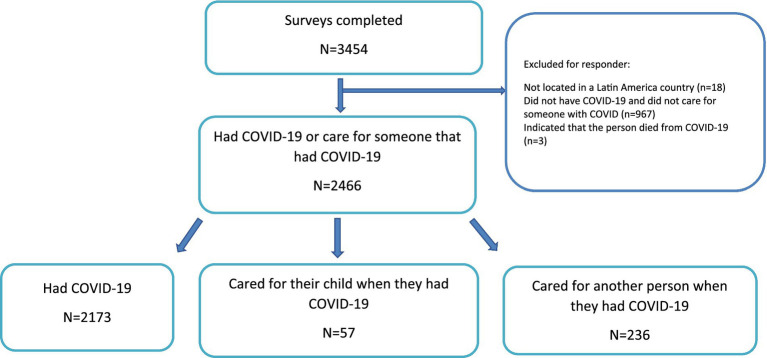
Flow diagram.

A total of 3,454 responses were collected. Of these, 2,466 people reported having COVID-19 (Figure 1). Responders with COVID-19 were mainly females (65.9%) from 16 countries in LATAM, but the majority (97%) reported Ecuador, Mexico, Argentina, Colombia, Peru and Chile as their place of residence (Supplementary Figure 1). 1,178 (48%) of the responders had COVID-19 or cared for someone that had COVID-19 symptoms ≥3 months after infection. The main symptoms reported during the COVID-19 infection included extreme fatigue, headaches, and issues with pain or discomfort; while extreme fatigue and headaches were also the main symptoms reported in participants with LCC, the third most common complaint in this group being issues with concentration, thinking, and memory (Supplementary Table 1).

### Characteristics of the participants with LCC

Participants with LCC were more likely (*p* < 0.001) to have COVID-19 infection earlier in the pandemic (Jan 2020–Jun 2021) than in the subsequent waves (Jul 2021-Sep 2022; Supplementary Figure 2), and reported significantly (*p* < 0.001) more COVID-19 symptoms (mean 11.2 ± 4.8 vs. 7.9 ± 4.9) during the infectious period. They were significantly older, a higher proportion of them had no COVID-19 vaccines, had more comorbidities, and needed supplementary oxygen therapy at the time of infection than participants without LCC (Table 1). A logistic regression model adjusted for participants’ characteristics and healthcare used during COVID-19 infection identified higher odds of having LCC in females, unvaccinated people, smokers, and people who visited the PCP, ED, or were hospitalized (Table 2). Participants with LCC that completed their surveys for themselves (*n* = 1,036) expressed having a good health on that day (mean 7.26 ± 1.6).

**Table 1:** Characteristics of survey respondents with and without long COVID condition (LCC).

Variable	Respondents with COVID-19 *N* = 2466*	No LCC symptoms *N* = 1,211	LCC symptoms *N* = 1,178	*p*
Gender (*n*, % female)	1,626 (65.9%)	782 (64.7%)	803 (68.2%)	0.07
Age, mean (SD)	39.5 (53.3)	35.6 (14.4)	37.9 (14.9)	<0.001
Age group (*n*, %)				
<19	200 (8.1%)	106 (8.8%)	83 (7.1%)	<0.001
20–29	736 (29.8%)	401(33.2%)	315 (26.8%)	
30–39	556 (22.5%)	274 (22.6%)	267 (22.7%)	
40–49	478 (19.4%)	210 (17.3%)	257 (21.9%)	
50–59	295 (12.0%)	133 (11.0%)	154 (13.1%)	
60–69	130 (5.3%)	64 (5.3%)	60 (5.1%)	
70+	71 (2.9%)	21 (1.7%)	38 (3.2%)	
COVID test (*n*, % yes)	2098 (85.0%)	999 (83.0%)	984 (84.2%)	0.43
COVID vaccines at the time of infection				
0	926 (37.6%)	388 (32.1%)	470 (40.1%)	<0.001
1	239 (9.7%)	114 (9.4%)	108 (9.2%)	
2	622 (25.2%)	312 (25.8%)	255 (21.7%)	
>=3	799 (32.4%)	393 (32.6%)	340 (29.0%)	
Smoking				
No	1741 (70.6%)	901 (75.3%)	792 (68.0%)	<0.001
Former	452 (18.3%)	181 (15.1%)	256 (22.0%)	
Yes	237 (9.6%)	114 (9.5%)	116 (10.0%)	
Main health conditions at the time of COVID-19				
None	1799 (73.0%)	957 (79.1%)	791 (67.1%)	<0.001
Respiratory disorders	134 (5.4%)	39 (3.2%)	87 (7.4%)	<0.001
Cardiovascular disorders	172 (7.0%)	75 (6.2%)	90 (7.6%)	0.16
Diabetes	77 (3.1%)	29 (2.4%)	47 (4.0%)	0.03
Obesity	111 (4.5%)	47 (3.9%)	62 (5.3%)	0.11
Other metabolic disorders	93 (3.8%)	32 (2.6%)	59 (49.3%)	0.01
Rheumatic/autoimmune disorders	27 (1.1%)	14 (1.2%)	12 (1.0%)	0.74
Mental health disorders	5 (0.2%)	1 (0.1%)	3 (0.3%)	0.30
Other	42 (1.7%)	15 (1.2%)	23 (2.0%)	
Used oxygen during COVID-19, yes (%)	272 (11.0%)	72 (5.9%)	176 (14.9%)	<0.001
Education (*n*, %)				
High school or less	562 (22.8%)	272 (22.5%)	267 (22.7%)	0.24
Apprenticeship	329 (13.3%)	177 (14.6%)	142 (12.1%)	
College/some university	71 (2.9%)	41 (3.4%)	29 (2.5%)	
University degree	1,041 (42.2%)	501 (41.4%)	509 (43.2%)	
Post-graduate degree	418 (17.0%)	196 (16.2%)	212 (18.0%)	
Prefer not to answer	45 (1.8%)	24 (2.0%)	19 (1.6%)	
Annual family income (*n*, %)				
<$3,000	683 (27.7%)	329 (27.2%)	328 (27.8%)	0.62
$3,000–5,000	314 (12.7%)	147 (12.1%)	165 (14.0%)	
$5,001–10,000	252 (10.2%)	124 (10.2%)	122 (10.4%)	
≥$10,001	329 (13.3%)	169 (14.0%)	152 (12.9%)	
Prefer not to answer	888 (36.0%)	442 (36.5%)	411 (34.9%)	

* Includes people who had COVID <3 months ago and responded “prefer not to answer” to the question about COVID symptoms ≥3 months (n = 77). Survey questions were not mandatory, and some participants did not provide a response to all questions. Other metabolic disorders = kidney, liver, thyroid problems, hyperlipidemias/hypercholesterolemia. Other = neurologic conditions, vertigo, cancer, gastric problems, etc.

**Table 2:** Factors associated with reported long COVID condition.

Variables	Univariable		Multivariable	
	LCC yes		LCC yes	
	OR (CI)	*p*	OR (CI)	*p*
Sex (Ref M)				
Females	1.17 (0.9–1.4)	0.07	1.28 (1.1–1.5)	0.01
Age groups (Ref ≤ 19 years)				
20–29	1.00 (0.7–1.4)	0.98	1.05 (0.7–1.5)	0.77
30–39	1.24 (0.8–1.7)	0.19	1.26 (0.8–1.8)	0.21
40–49	1.56 (1.1–2.2)	0.01	1.47 (1.0–2.1)	0.04
50–59	1.48 (1.0–2.1)	0.04	1.44 (1.0–2.1)	0.08
60–69	1.19 (0.7–1.9)	0.43	1.03 (0.6–1.7)	0.91
≥70	2.31 (1.3–4.2)	<0.01	1.21 (0.6–2.4)	1.03
COVID-19 vaccine (Ref none)				
1	0.78 (0.6–1.1)	0.10	0.82 (0.6–1.1)	0.24
2	0.68 (0.5–0.8)	<0.001	0.75 (0.6–0.9)	0.01
≥3	0.71 (0.6–0.8)	<0.001	0.81 (0.6–1.0)	0.05
Number of COVID-19 symptoms during infection	1.14 (1.1–1.2)	<0.001	1.13 (1.1–1.2)	<0.001
Smoking (Ref No)				
Former	1.16 (0.8–1.5)	0.29	1.30 (0.9–1.7)	0.09
Yes	1.61 (1.3–1.9)	<0.001	1.52 (1.2–1.9)	0.01
Main health conditions at the time of COVID-19 (Ref No)				
None	0.41 (0.1–2.3)	0.31	0.69 (0.1–4.1)	0.67
Respiratory disorders	1.12 (0.2–6.3)	0.90	1.68 (0.3–10.6)	
Cardiovascular disorders	0.60 (0.1–3.4)	0.56	0.82 (0.1–5.1)	0.58
Diabetes	0.81 (0.1–4.7)	0.82	0.97 (0.1–6.2)	0.84
Obesity	0.66 (0.1–3.7)	0.64	0.96 (0.1–6.0)	0.97
Other metabolic disorders	0.92 (0.1–5.3)	0.93	1.18 (0.2–7.5)	0.97
Rheumatic/autoimmune disorders	0.43 (0.1–2.7)	0.37	0.58 (0.1–4.2)	0.86
Mental health disorders	1.50 (0.1–25.3)	0.78	1.11 (0.1–20.2)	0.94
Other	0.76 (0.1–4.7)	0.77	1.30 (0.2–8.9)	0.79
Used oxygen during COVID-19 (Ref No)				
Yes	2.78 (2.0–3.7)	<0.001	0.98 (0.6–1.4)	0.94
Visited the primary care provider during COVID-19 (Ref No)				
Yes	1.71 (1.5–2.0)	<0.001	1.27 (1.0–1.5)	0.02
Visited the emergency department during COVID-19 (Ref No)				
Yes	2.09 (1.7–2.5)	<0.001	1.28 (1.0–1.6)	0.04
Hospitalized during COVID-19 (Ref No)				
Yes	3.15 (2.3–4.3)	<0.001	1.76 (1.1–2.7)	0.01

### Activity limitations

A third of participants with LCC (33%) expressed that they had to decrease the time regularly spent at work, school and other usual activities (Table 3). A higher percentage of these patients were male, older, had none or one COVID-19 vaccine, had a higher prevalence of respiratory and metabolic disorders and/or diabetes, had a higher number of COVID symptoms and required supplemental oxygen during the COVID-19 infection, and used more health services than their counterparts (Supplementary Table 2). An adjusted model found that LC participants who reduced their activities had persistent difficulty sleeping (OR 1.90, 1.29–2.78), chest pain with activity (OR 1.82, 1.20–2.76), depression (OR 1.58, 1.06–3.34), and problems with concentration, thinking, and memory (OR 1.46, 1.03–2.08; Table 4).

**Table 3:** Characteristics of participants with long COVID condition and impact on their activities (*n* = 1,178).

Variable	Decreased time spent at work, school, and other activities			Needed help with ADL		
	Yes	No	*p*	Yes	No	*p*
	*N* = 338	*N* = 815		*N* = 91	*N* = 1,075	
Gender (*n*, % female)	216 (63.9%)	571 (70.1%)	0.04	55 (60.4%)	742 (69.1%)	0.09
Age, mean (SD)	41.0 (16.8)	36.8 (13.7)	<0.001	44.1 (19.8)	37.4 (14.2)	<0.001
Age group (n, %)						
<19	25 (7.4%)	56 (6.9%)	<0.001	6 (6.7%)	76 (7.1%)	<0.001
20–29	78 (23.1%)	227 (28.0%)		19 (21.1%)	292 (27.2%)	
30–39	55 (16.3%)	207 (25.5%)		17 (18.9%)	248 (23.1%)	
40–49	81 (24.0%)	173 (21.3%)		17 (18.9%)	237 (22.1%)	
50–59	53 (15.7%)	98 (12.1%)		9 (10.0%)	144 (13.4%)	
60–69	27 (8.0%)	32 (3.9%)		11 (12.2%)	49 (4.6%)	
70+	18 (5.3%)	19 (2.3%)		11 (12.2%)	26 (2.4%)	
COVID vaccines at the time of infection						
0	160 (47.6%)	303 (37.3%)	<0.01	34 (37.8%)	433 (40.4%)	0.87
1	33 (9.8%)	71 (8.7%)		10 (11.1%)	93 (8.7%)	
2	66 (19.6%)	183 (22.5%)		20 (22.2%)	232 (21.7%)	
>=3	77 (22.9%)	256 (31.5%)		26 (28.9%)	313 (29.2%)	
Number of COVID-19 symptoms during infection	13.4 (4.1)	10.3 (4.7)	<0.001	12.8 (5.1)	11.0(4.7)	<0.001
Smoking						
No	220 (65.9%)	557 (69.1%)	0.56	58 (64.4%)	726 (68.3%)	0.69
Former	78 (23.4%)	171 (21.2%)		21 (23.3%)	233 (21.9%)	
Yes	36 (10.8%)	78 (9.7%)		11 (12.2%)	104 (9.8%)	
Main health conditions at the time of COVID-19						
None	189 (55.9%)	586 (71.9%)	<0.001	40 (44.0%)	742 (69.0%)	<0.001
Respiratory disorders	36 (10.7%)	49 (6.0%)	0.01	18 (19.8%)	69 (6.4%)	<0.001
Cardiovascular disorders	33 (9.8%)	55 (6.7%)	0.08	10 (11.0%)	78 (7.3%)	0.19
Diabetes	22 (6.5%)	23 (2.8%)	<0.01	8 (8.8%)	38 (3.5%)	0.02
Obesity	22 (6.5%)	39 (4.8%)	0.23	5 (5.5%)	57 (5.3%)	0.93
Other metabolic disorders	20 (5.9%)	38 (4.7%)	0.38	6 (6.6%)	53 (4.9)	0.48
Rheumatic/autoimmune disorders	7 (2.1%)	5 (0.6%)	0.03	1 (1.1%)	11 (1.0%)	0.94
Mental health disorders	2 (0.6%)	1 (0.1%)	0.16	0	3 (0.3%)	0.61
Other	7 (2.1%)	15 (1.8%)	0.79	2 (2.2%)	21 (2.0%)	0.87
Used oxygen during COVID-19, yes (%)	101 (29.9%)	72 (8.8%)	<0.001	41 (45.1%)	133 (12.4%)	<0.001
Healthcare use during COVID-19						
Primary Care	165 (48.8%)	163 (20.0%)	<0.001	42 (46.2%)	288 (26.8%)	<0.001
Emergency department	85 (25.1%)	39 (4.8%)	<0.001	31 (34.1%)	95 (8.8%)	<0.001
Hospital	36 (10.7%)	12 (1.5%)	<0.001	18 (19.8%)	31 (2.9%)	<0.001
Specialist	200 (59.2%)	198 (24.3%)	<0.001	56 (61.5%)	344 (32.0%)	<0.001
Physical therapist	86 (26.1%)	66 (8.2%)	<0.001	31 (36.9%)	121 (11.4%)	<0.001
Occupational therapist	24 (7.3%)	12 (1.5%)	<0.001	8 (9.5%)	28 (2.6%)	<0.001
Respiratory therapist	86 (26.1%)	84 (10.4%)	<0.001	29 (34.5%)	142 (13.4%)	<0.001
Speech pathologist	8 (2.4%)	5 (0.6%)	0.01	5 (6.0%)	8 (0.8%)	<0.001
Physiologist	84 (25.5%)	78 (9.7%)	<0.001	15 (17.9%)	146 (13.7%)	0.29

Survey questions were not mandatory, and some participants did not provide a response to all questions. Other metabolic disorders = kidney, liver, thyroid problems, hyperlipidemias/hypercholesterolemia. Other = neurologic conditions, vertigo, cancer, gastric problems, etc.

**Table 4:** Factors associated with greater activity limitations in long COVID condition (LCC).

	Decreased time spent at work, school, and other activities (yes/no)	Needed help with ADLs (yes/no)
Variables	Adjusted	Adjusted	OR (CI)	OR (CI)
Sex (Ref M)		
Females	**0.63 (0.45–0.86)**	0.93 (0.56–1.54)
Age	**1.02 (1.01–1.03)**	**1.03 (1.01–1.04)**
LCC symptoms		
Extreme fatigue	1.32 (0.85–2.04)	0.79 (0.34–1.82)
Headaches	0.99 (0.69–1.43)	0.83 (0.40–1.73)
Issues with pain or discomfort	0.87 (0.55–1.35)	1.14 (0.44–2.92)
Muscle or joint pain	1.31 (0.74–1.74)	1.29 (0.52–3.20)
General muscle weakness	1.29 (0.84–1.97)	0.44 (0.17–1.14)
Cough/noisy breathing	0.88 (0.62–1.79)	1.49 (0.78–2.86)
SOB with activity	1.22 (0.83–1.78)	1.17 (0.51–2.69)
Difficulty sleeping	**1.90 (1.29–2.78)**	0.72 (0.33–1.52)
Anxiety	1.12 (0.76–1.67)	1.18 (0.54–2.58)
Issues with concentration, thinking, and memory	**1.46 (1.03–2.08)**	0.73 (0.35–1.52)
SOB at rest	1.35 (0.87–2.09)	**2.95 (1.33–6.54)**
Chest pain at activity	**1.82 (1.20–2.76)**	0.86 (0.38–1.94)
Difficulty eating, drinking, and swallowing	0.76 (0.48–1.20)	1.93 (0.94–3.97)
Chest pain at rest	0.73 (0.45–1.19)	1.28 (0.55–2.97)
Depression	**1.58 (1.06–2.34)**	0.92 (0.43–1.92)
Difficulty walking	1.45 (1.26–3.38)	**3.32 (1.55–7.10)**
Difficulty controlling movement of the body	0.94 (0.591.49)	1.82 (0.86–3.86)
Dizziness, faint, and loss of consciousness	1.03 (0.69–1.54)	1.17 (0.60–2.28)
Other symptoms	**2.07 (1.26–3.38)**	0.98 (0.38–2.49)

Survey questions were not mandatory, and some participants did not provide a response to all questions. Other symptoms: change in taste and smell, congestion, gastro-intestinal symptoms, runny nose, rapid or irregular heart rate.

8% of the participants needed help with ADLs, such as personal hygiene or grooming, dressing, toileting, transferring or ambulating, and eating due to LCC (Table 3). These were significantly older, with a higher number of COVID-19 symptoms during infection. A higher proportion of them had respiratory disorders and diabetes, needed supplementary oxygen during COVID-19 infection, and used various healthcare services due to LCC (Supplementary Table 2). An adjusted model found that LCC participants who needed help with ADLs were significantly more likely to have difficulty walking (OR 3.32, 1.55–7.10), and shortness of breath (SOB) at rest (OR 2.95, 1.33–6.54; Table 4).

Among the participants who self-reported LCC, those who reduced their usual activities (mean 6.5 ± 1.9 vs. 7.6 ± 1.5, *p* < 0.001), as well as those who needed help with their ADLs (mean 5.8 ± 2.5 vs. 7.3 ± 1.6, *p* < 0.001), reported poorer health on that day compared to participants without activity limitations.

### Healthcare use

During the COVID-19 infection, 60% of the participants consulted their PCP, 25% visited the ED, and 8% of the participants were hospitalized (Supplementary Table 3). The main symptoms presented by the participants who used these services included extreme fatigue, headaches, issues related to pain or discomfort, and generalized muscle weakness.

Due to LCC symptoms, 33% of the participants consulted their PCP, 13% visited the ED, and 5% were hospitalized (Supplementary Table 3). The top symptoms reported by these healthcare users included extreme fatigue, headaches, SOB with activity, and difficulty sleeping. The main complaints in this group of participants (35%) who sought a specialist included fatigue, sleep difficulties, headaches, muscle or joint pain, and SOB with activity. 32% of the participants consulted one or more therapists. The most consulted therapists were respiratory therapists (15%) and psychologists (14%), followed by physical therapists (13%), occupational therapists (3%), and speech pathologists (1%).

About half of the participants who used the services of specialists (50%) or therapists (47%) stated that they reduced the time they regularly spent on their usual activities, and 14% needed help with their ADLs due to LCC.

## Discussion

Almost half (48%) of the participants had LCC symptoms in our study. This finding aligns with evidence from recent studies that reported LCC prevalence in a couple of South American countries [50% in Colombia (19) and 63% in Brazil (20)]. Similar to other studies, higher odds of LCC were found in females (21, 22), smokers (23), and middle-aged subjects (24) with a higher number of COVID-19 symptoms (25) who reported using more healthcare services, suggesting severe illness during the infectious period (26–30). Participants with LCC were more likely to have had COVID-19 infection earlier in the pandemic, which aligns with existent evidence suggesting than individuals infected with Omicron are less likely to experience severe long COVID symptoms (31). In addition, our results showed that fully vaccine patients (two doses) were less likely to have LCC compared with unvaccinated or partially vaccinated subjects. These results are consistent with those observed in a recent meta-analysis that found a protective effect of vaccination in patients vaccinated with two doses (RR = 0.83, 95% CI: 0.74–0.94, *p* < 0.01), but not with a dose (RR = 0.83, 95% CI: 0.65–1.07, *p* = 0.14) (20). This meta-analysis reported that vaccination reduces the risk of cognitive dysfunction/symptoms, myalgia, and sleeping disorders, which are some of the predominant symptoms found in our study population. Furthermore, vaccination was effective against LCC in patients vaccinated before (RR = 0.82, 95% CI: 0.74–0.91, *p* < 0.01) or after SARS-CoV infection (RR = 0.83, 95% CI: 0.74–0.92, *p* < 0.01) (32, 33).

We found that a high proportion of patients with LCC had pre-existent respiratory diseases, diabetes, and other metabolic diseases. This aligns with the literature showing that comorbidities such as lung disease, diabetes, obesity, and organ transplantation are potential risk factors for LCC (21). Although the prevalence of respiratory diseases, specifically COPD, in LATAM was similar to the reported globally, considerable variability (34) was reported by country, probably explained by differences in smoking levels, industrialization, genetic factors, and other predisposing factors such as tuberculosis or asthma. Diabetes prevalence has increased over time in LATAM (22), to the point that the diabetes-associated mortality risk is higher than in any other world region (23) and it is currently a significant threat to health systems, economy, and population health (24, 25). Cardiovascular and metabolic diseases are also a big problem in LATAM (35), and the global burden of high body mass index (BMI) is well-established and quantified in low- and middle-income countries (36). Overall, it is important to consider that these comorbidities are highly prevalent in the region and, therefore, can potentially contribute to increasing the risk of LCC in these populations.

COVID-19 and LCC have a wide range of symptoms, which appear to fluctuate throughout the course of infection. There are no diagnostic tests to confirm LCC, therefore, clinicians mostly rely on symptoms to identify this condition. Consistent with literature, the most common LCC symptoms reported in our study were extreme fatigue, cognitive dysfunction, sleeping difficulties, and anxiety (26–28, 37). Headache was frequently reported in the study, although this symptom is less commonly described in the literature (28). Conversely, participants experienced less shortness of breath, cough, depression, and chest pain compared to other studies (26–28, 37).

Our data shows that one-third of LCC participants have not returned to their regular activities and 8% required help to complete their ADLs ≥3 months after infection. It has been observed that LCC affects patients’ physical function, ability to return to work, school or other regular activities, and impacts their health-related quality of life (8, 38). However, to the best of our knowledge, this is the first study to identify that activity limitations were more likely to be reported by men and older participants. In older adults, particularly those who are frail, their ADL may be affected to the point that help from a caregiver may be necessary (8). Activity limitations can have a detrimental economic effect on affected families due to loss of income and/or additional care costs. This burden could deeply impact LATAM countries, since family income is lower than in developed nations, government support is minimal or non-existent, and care is often provided by an immediate family member.

Another critical aspect to consider is the demand for health services by patients with LCC. Worldwide, primary care was essential in diagnosing and treating COVID-19, promoting compliance with protective measures, and reducing the demand for hospital services (39). These care units have also been shown to be relevant in the care of patients with LCC (40). In countries such as Austria and Germany, all patients with LCC are referred to the general practitioner for clinical evaluation regardless of whether or not they were hospitalized during their episode of active infection (41). In our study, approximately one-third of the participants sought a PCP, specialist, and/or therapists due to LCC symptoms, especially those who experienced activity limitations. It is important to recognize that differences in referral systems, insurance coverages, and healthcare policies across countries may have restricted access to some healthcare services and therefore affected the proportion of patients who were able to consult these healthcare professionals. Preliminary evidence suggests that most COVID-related care should be provided in primary care (36); however, it must be acknowledged that PCPs are challenged to provide care to these patients who often have a wide range of symptoms and are unlikely to respond to a single intervention. Therefore, it is essential to promote the need for interdisciplinary healthcare teams to manage this condition (42). Although in the current scenario, the increased requirements for specialized health services may raise costs for already strained health systems, and rehabilitation services in the region have been impacted by operational and infrastructure adjustments needed for compliance with the biosafety regulations brought on by the pandemic (43). Alternatives to these challenges should be explored since, according to our results, a significant proportion of patients with LCC symptoms will require an integrated approach to regain their health.

### Strengths and limitations

To our knowledge, our study is the first of its kind to provide comprehensive information about characteristics, symptoms, impact on patients’ activities, and healthcare access of patients who experience LCC in LATAM. Our results are also based on a large sample of people from LATAM unlike previous studies of specific cities. Also, online data collection enabled the research team to reach a large number of participants from all regions of LATAM as well as to minimize data entry errors. Nonetheless, some study limitations are worth noting. The questionnaire was completely anonymous and no personal identifiers such as names, emails, or IP addresses were collected. Furthermore, after the form was submitted, the participant was unable to make any changes to their responses. However, the survey was immediately made available again so that the respondent could fill it out for another family member. Therefore, the IP address of the participant’s computer could not be used to identify potential duplicate entries from the same user, which may have contributed to potential bias. Due to the non-probability convenience sampling method, the sample was not drawn randomly from the population of interest to ensure representativeness. Therefore, these findings cannot be generalized to unrepresented countries, nor can they be used in any way to calculate the prevalence of LCC. People who use social media (and therefore were able to access the survey) could have different characteristics to those who do not use such platforms. Although we tried to reach people from other sources such as a list of patients, the online recruitment strategy and questionnaire administration could explain the oversampling of women in our study, since women tend to use Facebook and work in online environments more often than men. Some people may have been unintentionally excluded from the survey, as they may have limited or no access to a technological device and/or the Internet to complete the virtual questionnaire. Additionally, people with more symptoms or more severe symptoms may be more likely to respond to the survey. There is also the possibility of recall bias in this survey, as the data during COVID-19 (acute stage) was collected retrospectively.

## Conclusion

Our results supported previous findings in terms of the main characteristics and symptoms of patients experiencing LCC, identified characteristics of these patients who reported activity limitations, and described symptoms commonly reported among healthcare users in LATAM. This information is valuable to inform future service planning and resource allocation in alignment with the needs of this population.

## Data availability statement

The original contributions presented in the study are included in the article/Supplementary material, further inquiries can be directed to the corresponding author.

## Ethics statement

This study was approved by the University of Manitoba ethics committee (HS25587/H2022:230). Before completing the survey, the participants were informed that by clicking next and advancing to the next page, they gave their consent to participate in the study.

## Author contributions

DS-R conceived the study, organized the database, and performed the statistical analysis. AA-F, RT-C, VB-C, SC, MM-S, BL-H, RS-P, SL, and DS-R helped with survey design, refinement, and distribution, and supported the data collection process. AA-F, RT-C, VB-C, and DS-R wrote the first draft of the manuscript. All authors contributed to the article and approved the submitted version.

## Conflict of interest

The authors declare that the research was conducted in the absence of any commercial or financial relationships that could be construed as a potential conflict of interest.

## Publisher’s note

All claims expressed in this article are solely those of the authors and do not necessarily represent those of their affiliated organizations, or those of the publisher, the editors and the reviewers. Any product that may be evaluated in this article, or claim that may be made by its manufacturer, is not guaranteed or endorsed by the publisher.
