# Supplementary material for: Exploring long COVID condition in Latin America: Its impact on patients’ activities and associated healthcare use
Source: Front Med (Lausanne). 2023 Apr 20;10:1168628. doi: 10.3389/fmed.2023.1168628 (PMC10157152; doi:10.3389/fmed.2023.1168628)
Supplement: Supplementary file 1 [file Data_Sheet_1.docx]

Supplementary material

Figures

**Supplemental Figure 1.** COVID-19 Cases by country of residence


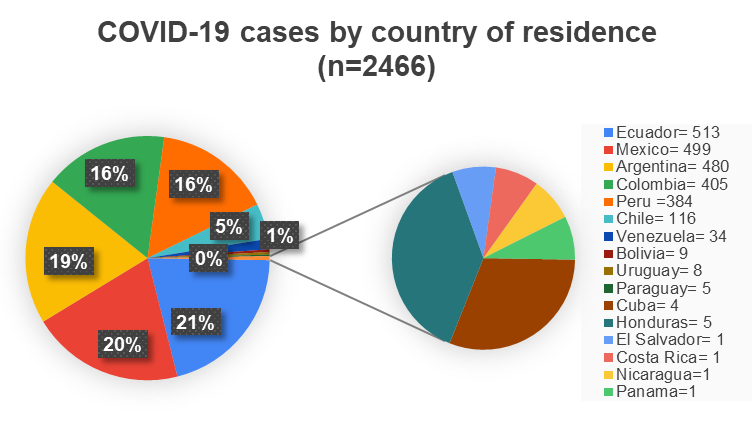


**Supplemental Figure 2.** Long COVID condition by date of infection

Tables

| **Supplemental Table 1.** Symptoms experienced in participants with and without Long COVID condition (LCC). | | | | | |
| --- | --- | --- | --- | --- | --- |
| **Symptoms, n (%)** | COVID-19 symptoms (acute phase) | | | | LCC  symptoms  n=1178 |
|  | **All***  N=2389 | LCC-No  N=1211 | LCC-Yes  N=1178 | *p* |  |
| Extreme Fatigue | **1935 (81.0%)** | 894 (73.8%) | 1041 (88.4%) | <0.001 | **746 (63.3%)** |
| Headaches | **1916 (80.2%)** | 923 (76.2%) | 993 (84.3%) | <0.001 | **640 (54.3%)** |
| Issues with pain or discomfort | **1835 (76.8%)** | 851 (70.3%) | 984 (83.5%) | <0.001 | 478 (40.6%) |
| Muscle or joint pain | 1700 (71.2%) | 768 (63.4%) | 932 (79.1%) | <0.001 | 581 (45.8%) |
| General muscle weakness | 1666 (69.7%) | 741 (61.2%) | 925 (78.5%) | <0.001 | 539 (45.8%) |
| Cough / noisy breathing | 1548 (64.8%) | 707 (58.4%) | 841 (71.4%) | <0.001 | 426 (36.2%) |
| SOB with activity | 1383 (57.9%) | 561 (46.3%) | 822 (69.8%) | <0.001 | 549 (46.6%) |
| Difficulty sleeping | 1290 (54.0%) | 533 (44.0%) | 757 (64.3%) | <0.001 | 575 (48.8%) |
| Anxiety | 1181 (49.4%) | 432 (35.7%) | 749 (63.6%) | <0.001 | 565 (48.0%) |
| Issues with concentration, thinking and memory | 1072 (44.9%) | 369 (30.5%) | 703 (65.6%) | <0.001 | **589 (50.0%)** |
| SOB at rest | 994 (41.6%) | 378 (31.2%) | 616 (52.3%) | <0.001 | 341 (28.9%) |
| Chest pain at activity | 967 (40.5%) | 351 (29.0%) | 616 (52.3%) | <0.001 | 380 (32.3%) |
| Difficulty eating, drinking, and swallowing | 933 (39.1%) | 399 (32.9%) | 534 (45.3%) | <0.001 | 253 (21.5%) |
| Chest pain at rest | 914 (38.3%) | 345 (28.5%) | 569 (48.3%) | <0.001 | 287 (24.4%) |
| Depression | 894 (37.4%) | 321 (26.5%) | 573 (48.6%) | <0.001 | 447 (37.9%) |
| Difficulty walking | 861 (36.0%) | 301 (24.9%) | 560 (47.5%) | <0.001 | 293 (24.9%) |
| Difficulty controlling movement of the body | 572 (23.9%) | 207 (63.2%) | 365 (31.0%) | <0.001 | 252 (21.4%) |
| Dizziness, faint, Loss of consciousness | 499 (20.9%) | 158 (13.0%) | 341 (28.9%) | <0.001 | 270 (22.9%) |
| Other: |  |  |  |  |  |
| Change in taste and smell | 266 (11.1%) | 129 (10.7%) | 137 (11.6%) | 0.45 | 45 (3.8%) |
| Congestion | 46 (1.9%) | 20 (1.7%) | 26 (2.2%) | 0.32 | 5 (0.4%) |
| Gastro-intestinal symptoms | 91 (3.8%) | 37 (3.1%) | 54 (4.6%) | 0.05 | 7 (0.6%) |
| Runny nose | 10 (0.4%) | 5 (0.4%) | 5 (0.4%) | 0.96 | 6 (0.5%) |
| Fever | 137 (5.7%) | 78 (6.4%) | 59 (5.0%) | 0.13 | - |
| Rapid or irregular heart rate | 22 (0.9%) | 5 (0.4%) | 17 (1.4%) | 0.01 | 16 (1.4%) |
| Rash | 5 (0.2%) | 2 (0.2%) | 3 (0.3%) | 0.63 | - |
| *Excludes people who had COVID <3 months ago and responded “prefer not to answer” to the question about COVID symptoms ≥3 months (n=77). *Questions were not mandatory, not all respondents responded to each question.* ***Bold****= top 3 symptoms.* | | | | | |

| **Supplemental Table 2.** Characteristics of LCC participants and activity limitations n=1178 | | | | | | |
| --- | --- | --- | --- | --- | --- | --- |
|  | **Decreased time spent at work, school, and other activities** | | | **Needed help with ADLs** | | |
| **LCS symptom** | Yes  N=388 | No  N=815 | *p* | Yes  N=91 | No  N=1075 | *p* |
| Extreme Fatigue | 280 (82.8%) | 451 (55.3%) | <0.001 | 72 (79.1%) | 666 (62.0%) | 0.001 |
| Headaches | 238 (70.4%) | 386 (47.4%) | <0.001 | 67 (73.6%) | 566 (52.7%) | <0.001 |
| Issues with pain or discomfort | 204 (60.4%) | 261 (32.0%) | <0.001 | 65 (71.4%) | 407 (37.9%) | <0.001 |
| Muscle or joint pain | 240 (71.0%) | 327 (40.1%) | <0.001 | 71 (78.0%) | 502 (46.7%) | <0.001 |
| General muscle weakness | 229 (67.8%) | 297 (36.4%) | <0.001 | 65 (71.4%) | 466 (43.3%) | <0.001 |
| Cough / noisy breathing | 164 (48.5%) | 249 (30.6%) | <0.001 | 63 (69.2%) | 356 (33.1%) | <0.001 |
| SOB with activity | 223 (66.0%) | 312 (38.3%) | <0.001 | 72 (79.1%) | 471 (43.8%) | <0.001 |
| Difficulty sleeping | 251 (74.3%) | 311 (38.2%) | <0.001 | 65 (71.4%) | 503 (46.8%) | <0.001 |
| Anxiety | 229 (67.8%) | 320 (39.3%) | <0.001 | 64 (70.3%) | 496 (46.1%) | <0.001 |
| Issues with concentration, thinking and memory | 236 (69.8%) | 336 (41.2%) | <0.001 | 64 (70.3%) | 518 (48.2%) | <0.001 |
| SOB at rest | 158 (46.7%) | 170 (20.9%) | <0.001 | 66 (72.5%) | 269 (25.0%) | <0.001 |
| Chest pain at activity | 172 (50.9%) | 196 (24.0%) | <0.001 | 56 (61.5%) | 318 (29.6% | <0.001 |
| Difficulty eating, drinking, and swallowing | 112 (33.1%) | 128 (15.7%) | <0.001 | 56 (61.5%) | 192 (17.9%) | <0.001 |
| Chest pain at rest | 130 (38.5%) | 147 (18.0%) | <0.001 | 54 (59.3%) | 229 (21.3%) | <0.001 |
| Depression | 205 (60.7%) | 230 (28.2%) | <0.001 | 56 (61.5%) | 386 (35.9%) | <0.001 |
| Difficulty walking | 145 (42.9%) | 135 (16.6%) | <0.001 | 63 (69.2%) | 223 (20.7%) | <0.001 |
| Difficulty controlling movement of the body | 119 (35.2%) | 121 (14.8%) | <0.001 | 56 (61.5%) | 188 (17.5%) | <0.001 |
| Dizziness, faint, Loss of consciousness | 118 (34.9%) | 141 (17.3%) | <0.001 | 47 (51.6%) | 219 (20.4%) | <0.001 |
| Other symptoms | 37 (10.9%) | 57 (7.0%) | <0.001 | 6 (6.6%) | 88 (8.2%) | 0.59 |
| **Healthcare use due to LCS** | | | | | | |
| Primary Care | 165 (48.8%) | 163 (20.0%) | <0.001 | 45 (46.2%) | 288 (26.8%) | <0.001 |
| Emergency department | 85 (25.1%%) | 39 (4.8%) | <0.001 | 31 (34.1%) | 95 (8.8%) | <0.001 |
| Hospital | 36 (10.7%) | 12 (1.5%) | <0.001 | 18 (19.8%) | 31 (2.9%) | <0.001 |
| Specialist | 200 (59.2%) | 198 (24.3%) | <0.001 | 56 (61.5%) | 344 (32.0%) | <0.001 |
| Physical therapist | 86 (26.1%) | 66 (8.2%) | <0.001 | 31 (36.9%) | 121 (11.4%) | <0.001 |
| Occupational therapist | 24 (7.3%) | 12 (1.5%) | <0.001 | 8 (9.5%) | 28 (2.6%) | <0.001 |
| Respiratory therapist | 86 (26.1%) | 84 (10.4%) | <0.001 | 29 (34.5%) | 142 (13.4%) | <0.001 |
| Speech pathologist | 8 (2.4%) | 5 (0.6%) | 0.009 | 5 (6.0%) | 8 (0.8%) | <0.001 |
| Physiologist | 84 (25.5% | 78 (9.7%) | <0.001 | 15 (17.9%) | 146 (13.7%) | 0.29 |
| Long COVID condition (LCC). Survey questions were not mandatory, and some participants did not provide a response to all questions. Other symptoms: change in taste and smell, congestion, gastro-intestinal symptoms, runny nose, rapid or irregular heart rate. | | | | | | |

| **Supplemental Table 3. Healthcare use by symptoms** | | | | | | | | | | | | |
| --- | --- | --- | --- | --- | --- | --- | --- | --- | --- | --- | --- | --- |
|  | **COVID-19**  n=2466 | | | **Long COVID condition** (≥3 months after COVID-19)  n=1178 | | | | | | | | |
| **Symptoms** | **PCP** | **ED** | **Hospital** | **PCP** | **ED** | **Hospital** | **Specialist** | **Therapists** | | | | |
|  |  |  |  |  |  |  |  | **Physical therapist** | **Occupational therapist** | **Respiratory therapist** | **Speech therapist** | **Psychologist** |
|  | N=1486 (60%) | N=608  (25%) | N=205  (8%) | N=383  (33%) | N=158  (13%) | N=64  (5%) | N=528  (21%) | N=155  (13%) | N=36  (3%) | N=172  (15%) | N=13  (1%) | N=162  (14%) |
| Extreme Fatigue | **1258 (84.7%)** | **528 (86.8%)** | **181 (88.3%)** | **241 (62.9%)** | **104 (65.8%)** | **39 (60.9%)** | 290 (21.2%) | **114 (57.6%)** | **29**  **(76.3%)** | **126**  **(55.8%)** | 8  (53.3%) | **125**  **(66.8%)** |
| Headaches | **1221 (82.2%)** | **511 (84.0%)** | 163 (79.5%) | **204 (53.3%)** | 93  (58.8%) | 36 (56.3%) | **245 (46.4%)** | 101 (48.5%) | 26  (68.4%) | **104**  **(46.0%)** | 8  (53.3%) | 104  (55.6%) |
| Issues with pain or discomfort | **1197 (80.6%)** | **500 (82.2%)** | **171 (83.4%)** | 164 (42.8%) | 65 (41.1%) | 33 (51.6%) | 200 (37.9%) | 86 (43.4%) | 25  (65.8%) | 82  (36.3%) | 7  (46.7%) | 78  (417%) |
| General muscle weakness | 1108 (73.8%) | 471 (77.5%) | **171 (83.4%)** | 179 (46.7%) | 89 (56.3%) | 35 (54.7%) | 226 (42.8%) | 104 (52.5%) | **29**  **(76.3%)** | 92  (40.7%) | 8  (53.3%) | 82  (43.9%) |
| Muscle or joint pain | 1108 (74.6%) | 474 (78.0%) | 164 (80.0%) | 164 (42.8%) | 94 (59.5%) | 35 (54.7%) | **245 (46.4%)** | **111 (56.1%)** | 30  (78.9%) | 103  (45.6%) | **10**  **(66.7%)** | 96  (51.3%) |
| SOB with activity | 934 (62.9%) | 424 (69.7%) | 151 (73.7%) | 199 (52.0%) | **97 (61.4%)** | **43 (67.2%)** | 242 (45.8%) | 102 (51.5%) | **30**  **(78.9%)** | **125**  **(55.3%)** | 10  (66.7%) | 85  (45.5%) |
| Issues with concentration, thinking and memory | 720 (48.5%) | 337 (55.4%) | 126 (61.5%) | 198 (51.7%) | 81 (51.3%) | **38 (59.4%)** | 240 (45.5%) | 96 (48.5%) | 26  (68.4%) | 94  41.6%) | 7  (46.7%) | 111  (59.4%) |
| Cough / noisy breathing | 1011 (68.0%) | 436 (71.7%) | 156 (76.1%) | 146 (38.1%) | 76 (48.1%) | 34 (53.1%) | 183 (34.7%) | 74 (37.4%) | 25  (65.8%) | 88  (38.9%) | 6  (40.0%) | 53  (28.3%) |
| Difficulty sleeping | 871 (58.6%) | 385 (63.3%) | 144 (70.2%) | **206 (53.8%)** | **96 (60.8%)** | 29 (45.3%) | **251 (47.5%)** | **106 (53.6%)** | 28  (73.7%) | 107  (47.3%) | **10**  **(66.7%)** | **114**  **(61.0%)** |
| Anxiety | 794 (53.4%) | 369 (60.7%) | 137 (66.8%) | 193 (50.4%) | 88 (55.7%) | 35 (54.7%) | 235 (44.5%) | 93 (47.0%) | 26  (68.4%) | 94  (41.6%) | **10**  **(66.7%)** | **119**  **(63.6%)** |
| SOB at rest | 675 (45.4%) | 353 (58.1%) | 150 (73.2%) | 125 (32.6%) | 68 (43.0%) | 31 (48.4%) | 169 (32.0%) | 77 (38.9%) | 25  (65.8%) | 77  (34.1%) | 8  (53.3%) | 44  (23.5%) |
| Dizziness, faint, Loss of consciousness | 347 (23.4%) | 179 (29.4%) | 62 (30.2%) | 97 (25.3%) | 56 (35.4%) | 31 (48.4%) | 117 (22.2%) | 58 (29.3%) | 21  (55.3%) | 56  (24.8%) | 7  (46.77%) | 47  (25.1%) |
| Chest pain at activity | 666 (44.8%) | 325 (53.5%) | 122 (61.0%) | 137 (35.8%) | 74 (46.8%) | 33 (51.6%) | 162 (30.7%) | 67 (33.8%) | 25  (65.8%) | 78  (34.5%) | 6  (40.0%) | 56  (29.9%) |
| Depression | 616 (41.5%) | 289 (47.5%) | 108 (52.7%) | 153 (39.9%) | 66 (41.8%) | 29 (45.3%) | 190 (36.0%) | 81 (40.9%) | 24  63.2%) | 76  (33.6%) | 9  (60.0%) | 99  (52.9%) |
| Chest pain at rest | 634 (42.7%) | 308 (50.7%) | 125 (61.0%) | 100 (26.1%) | 60 (38.0%) | 32 (50.0.%) | 133 (25.2%) | 50 (25.3%) | 19  (50%) | 57  (25.2%) | 8  (53.3%) | 38  (20.3%) |
| Difficulty eating, drinking and swallowing | 621 (41.8%) | 294 (48.4%) | 117 (57.1%) | 87 (22.7%) | 57 (36.1%) | 30 (46.9%) | 107 (2.3%) | 54 (27.3%) | 16  (42.1%) | 53  (23.5%) | 8  (53.3%) | 31  (16.6%) |
| Difficulty walking | 585 (39.4%) | 313 (51.5%) | 131 (63.9%) | 116 (30.3%) | 65 (41.1%) | 33 (51.6%) | 141 (26.7%) | 69 (34.8%) | 23  (60.5%) | 70  (36.3%) | 8  (53.3%) | 45  (24.1%) |
| Difficulty controlling movement of the body | 376 (25.3%) | 199 (32.7%) | 93 (45.4%) | 88 (23.0%) | 57 (36.1%) | 30 (46.9%) | 113 (21.4%) | 56 (28.3%) | 16  (42.1%) | 53  (23.5%) | 7  (46.7%) | 43  (23.0%) |
| Other symptoms | 65 (4.3%) | 26  (4.3%) | 12  (5.99%) | 50 (13.0%) | 18  (11.4%) | 6  (9.3%) | 48  (9.0%) | 13  (8.3%) | 5  (13.9%) | 15  (8.7%) | 1  (7.7%) | 16  (9.9%) |
| PCP= Primary Care Provider, ED=Emergency Department. *Each person may have more than one symptom and may have used more than one service. Other symptoms: changes in taste and smell, congestion, gastro-intestinal symptoms, runny nose, rapid or irregular heart rate. **Bold**= top 3 symptoms. | | | | | | | | | | | | |
